# The Roche Cancer Genome Database 2.0

**DOI:** 10.1186/1755-8794-4-43

**Published:** 2011-05-17

**Authors:** Jan Küntzer, Daniela Maisel, Hans-Peter Lenhof, Stefan Klostermann, Helmut Burtscher

**Affiliations:** 1 Roche Diagnostics GmbH Pharma Research and Early Development Informatics, Penzberg, Germany; 2 Roche Diagnostics GmbH, Translational Research Sciences, Penzberg, Germany; 3 Center for Bioinformatics Saar, Saarland University, Saarbrücken, Germany; 4 Roche Diagnostics GmbH, Discovery Oncology, Penzberg, Germany

## Abstract

**Background:**

Cancer is a disease of genome alterations that arise through the acquisition of multiple somatic DNA sequence mutations. Some of these mutations can be critical for the development of a tumor and can be useful to characterize tumor types or predict outcome.

**Description:**

We have constructed an integrated biological information system termed the Roche Cancer Genome Database (RCGDB) combining different human mutation databases already publicly available. This data is further extended by hand-curated information from publications.

The current version of the RCGDB provides a user-friendly graphical interface that gives access to the data in different ways: (1) Single interactive search by genes, samples, cell lines, diseases, as well as pathways, (2) batch searches for genes and cell lines, (3) customized searches for regularly occurring requests, and (4) an advanced query interface enabling the user to query for samples and mutations by various filter criteria.

**Conclusion:**

The interfaces of the presented database enable the user to search and view mutations in an intuitive and straight-forward manner. The database is freely accessible at http://rcgdb.bioinf.uni-sb.de/MutomeWeb/.

## Background

Facilitated by the development of new high throughput techniques like SNP arrays the identification of genes involved in complex diseases such as cancer has made enormous progress over the recent years. This resulted in a huge amount of information on human genetic variations which have been described in various genes associated with a wide variety of diseases [1-3]. Sequence variations are being studied for a better understanding of the mechanism and development of cancer as a mutation-driven disease [4]. Furthermore, somatic mutations in cancer can be used in clinical studies and molecular pathology to characterize tumor types, to predict response to treatment, and to find the best suited treatment. Thus, mutation analysis can play an important role in drug discovery and personalized healthcare.

The proportion of mutations causally implicated in cancer is still unknown especially due to the high number of mutations between different tumors [5-8]. Although the number of unique variations for each cancer genome can be very high [9,10], only a few somatic variations will be critical for the development of the tumor. These causative variations, the so-called "drivers", are emerging because of selective pressure during tumorigenesis, whereas many mutations are only incidental or caused by genome instabilities, so-called "passengers" [11]. The differentiation of disease-causing driver mutations from passenger variations is a challenge for mutation analysis [12].

Despite the technological progress and increase of data, the benefit of this information is still limited due to the ever increasing number of databases [13] and the absence of a unified access to the mutation data. Currently, researchers are typically forced to browse several, often highly specialized databases to obtain the required information. A more complete understanding of relations and dependencies between mutations and cancer, however, requires the availability of an efficient integrative cancer genome information system.

## Methods

To facilitate the analyses of cancer mutations, we developed the second version of the *Roche Cancer Genome Database (RCGDB) *[14], an integrative biological information system combining different databases which are already publicly available. We use the concept of a data warehouse with a single database containing all information presented by the system. To this end, we developed an internal data model to store cancer genome information and implemented different importer routines to map external database information onto our model.

In the current version the RCGDB combines a huge variety of information on the human cancer genome. We integrated somatic mutations, which are not inherited but acquired during lifetime in somatic cells of an organism and might cause tissue specific tumors. This information is gathered from COSMIC at the Sanger Institute [15], the Cancer Genome Atlas project [16], the IARC TP53 database [17], OMIM [18], KinMutBase [19], and the L1CAM mutation database [20]. From the Cancer Genome Atlas we also integrated germline mutations which in contrast to the somatic mutations can be found in all cells of an organism including germ line cells. Furthermore, we imported additional data from publications, which we curated by hand. For both types of mutations, somatic and germline, we store all available sample information like tissue of origin and histology of the sample, or if the sample was a tumor cell line.

In addition to germline and somatic mutations, we retrieve SNPs and their corresponding frequencies from the international HapMap project [21]. We focus only on the HapMap data source for SNPs and decided not to integrate the dbSNP [22] since it contains too many false positive SNPs. The HapMap data is integrated as a third group of information, since for SNPs it is not known whether the sample information is derived from a tumor or a normal sample. Therefore, a correlation between diseases and SNPs cannot be calculated.

Furthermore, our system contains amplification data from the database of genomic variants and the NCBI SKY/M-FISH & CGH database [23], as well as pre-analyzed amplification data from the Tumorscape project at the BROAD institute [24].

The cancer genome data is further enriched by pathway information from KEGG [25], BioCarta http://www.biocarta.com and Roche internal networks.

## Results

A major aspect in designing the user interface was that users should be able to search and view mutations in an intuitive and straight-forward manner, without having to understand the architectural details of the warehouse system. To facilitate this, we grant access to the RCGDB via different ways:

### Simple Search

First, the database offers a Google-like web interface to search for cancer genome information on genes, samples, cell lines, diseases, as well as pathways. As a special feature each search is supported by an auto-suggestion functionality allowing, e.g. in the gene search, to query by NCBI GeneIDs, names, or synonyms.

**Figure 1:**
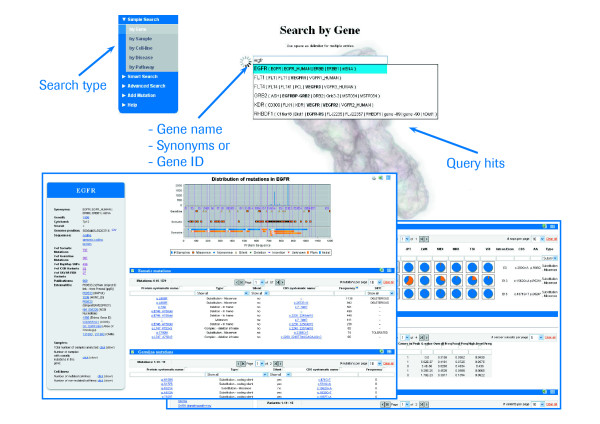
**Searching for cancer genome information of EGFR via the simple gene search interface**. The results of the query are shown in blue boxes in the lower part of the figure.

After performing a simple gene search the system presents a single page with all gene centered information. An example is shown in Figure 1. On the left-hand side, general gene information (symbol, alias, description, etc.) and mutation counts are presented in a card. The right-hand side shows an overview of all somatic and germline mutations based on the amino acid sequence of the gene including a histogram representing the sample frequencies. Below this overview separate sections each display a table of somatic mutations, germline mutations, SNPs, amplification data, CGH data, and SKY/M-FISH data. Each table features a filter functionality combined with an Excel export option.

For cell line queries we offer searches by cell line name or frequently used synonyms. The resulting page presents a card with general information on the sample (name, synonyms, tissue, histology, etc.) on the left-hand side. On the right-hand side, different sections display all known cancer genome information for the sample. This includes a list of all mutations and a table of all mutated genes. Below that we give an overview of all genes, which were found not to be mutated. For genes not mentioned in one of these two sections the database contains no mutation information for a given cell line.

In addition to the cell line search the RCGDB offers an interface to query samples by tissue and histology. The system will check all samples with the given filter for mutations and present all non-mutated and mutated genes in these samples. For each mutated gene we show the percentage of mutated samples, allowing to filter for the most mutated gene in the given tissue and histology.

Another possibility to query the system is given by the disease search interface based on OMIM disease names or IDs. After performing a search we present all genes associated with this disease by OMIM and all HapMap SNPs found in the region of these genes. These SNPs are not directly associated with the given disease but rather with a gene which is known to be associated with the disease.

For researchers working on special pathways we integrated KEGG and BioCarta pathways along with Roche internal pathways. After selecting the desired pathway we present a static pathway image where all genes are linked to the corresponding gene search page. For protein families within KEGG pathways the system will directly link to a gene batch search for all members of the family.

### Batch Search

The above-mentioned interfaces also allow batch searches for genes, samples and cell lines whereby multiple entries are separated by a single white space. As a result, on the left-hand side the system presents an overview of all matches found. If the search name is an alias, which can be unambiguously matched to a single gene or cell line, the corresponding match is shown. For ambiguous matches the user can refine the search by selecting the desired gene or sample. This directly updates the mutation findings presented on the right-hand side, as well as the section on mutated genes or mutated cell lines respectively (see Additional file 1).

### Smart Search

The second access point to the RCGDB are customized search interfaces (so called smart searches) for regularly occurring requests of higher complexity. The concept behind the smart searches are specialized interfaces for different applications, presenting the data in a highly condensed way. An example is the "mutation status" smart search as shown in Additional file 2. A classical and often occurring request from biologists refers to the mutation status of different genes in various cell lines. For each gene/cell line combination the researchers want to know if the gene is mutated or wildtype. For this question, we offer a smart query with separate input fields for genes and cell lines. Each input field has the same auto-suggestion features as known from the simple search interfaces and allows for multiple entries divided by spaces. In addition, each smart search interface presents a short description to help the user find the correct smart search fitting his purposes. As a result, the mutation status smart search presents a matrix with the genes as rows and cell lines as columns. For each gene/cell line combination the matrix cell shows if the gene is mutated (mut), wildtype (wt), or no information is known in the database (na). Cards on the left-hand side and right-hand side show if the cell line or gene was found in the system or not. This is especially useful if the user has pasted a number of genes or cell lines into the interface without using the auto-suggestion feature.

The smart searches are not only used for customized search interfaces but also for integrative analyses. An example for this is the pathway enrichment analysis query (see Additional file 2, lower panel), which for all KEGG pathways determines whether the number of mutated genes found in a subset of samples is statistically significant. The subset of samples is specified in the interface by the tissue and histology.

### Advanced Search

Furthermore, we included a powerful advanced search interface enabling the user to search for putative biomarkers. This interface allows to query for samples and mutations by various filter criteria, e.g. genes, samples, tissue, histology, or mutation, without any restriction in the number of filters. With the plus/minus sign one can add/remove single filters interactively. A logical operator can be selected for each filter allowing to add negative and "like" searches. The input fields show the same auto-suggestion features as known from the simple search interfaces, depending on the selected filter type. These fields also support multiple entries, which need to be separated by whitespace, as well as multiple and single character wildcards for "like" operators (see Additional file 3). After performing an advanced search all somatic mutations, germline mutations, and samples fulfilling the selected filter criteria are presented in separate sections at the bottom of the page. An additional statistics section shows a table with the counts of all matched amino acid mutations. For each mutation we also state the frequency of all samples, the frequency of all samples with at least one mutation, as well as the relative frequency at the given position.

## Discussion

In order to provide a central resource for scientists in the cancer research community, we developed the RCGDB as a comprehensive resource of cancer genome information. To date, a number of multi-gene locus-specific databases (LSDB) and single-gene LSDBs are publicly available and offer different kinds of human variation data by various interfaces. Unfortunately, the diversity of mutation information systems and the underlying data models make it difficult to mine human variation databases in an integrative approach. Thus for a comprehensive analysis of human variation data the researcher still has to search multiple databases.

These drawbacks have been overcome by the integrative concept underlying the RCGDB. The main idea here is to offer data spanning a variety of data sources and data types. The database not only offers the complete data available in the integrated data sources, but in addition includes mutation data from 14 publications which were curated by hand. To the best of our knowledge this data is currently not available in any one of the other databases. The huge benefit of this concept is illustrated in Table 1.

**Table 1:** Counts of the different mutations types grouped by source of origin

	Release	Somatic mutations	Germline mutations	SNPs	Genetic Variants (CGH)	Genetic Variants (SKY/M-FISH)
Catalogue of somatic mutations in cancer (COSMIC)	V43	19267	-	-	-	-
OMIM	-	2047	-	-	-	-
The Cancer Genome Atlas	MA files	18432	84113	-	-	-
IARC TP53 Database	R14	3942	-	-	-	-
KinMutBase	V3.0	565	-	-	-	-
University of Groningen L1CAM Mutation DB	1.0	206	-	-	-	-
International HapMap Project	27	-	-	1657504	-	-
Database of Genomic Variants	V9	-	-	-	85193	-
NCBI SKY/M-FISH & CGH Database	-	-	-	-	3681	6377
Publications	-	29159	5139	-	-	-
**Total (in RCGDB)**		73618	89252	1657504	88874	6377

Another important feature of the RCGDB compared to already published databases on human variation data can be found in our powerful search interfaces: Mutation databases offer mostly access via gene, sample and tissue search, which we extend by batch searches and additional pathway and OMIM disease searches. Our advanced filter search and especially our smart searches are very convenient and allow for full access to cancer genome data. Users whose primary interest lies within a specific biological problem will prefer the convenient Google-like web interface or customized smart searches providing powerful platform-independent visualization of the data. The search pages accept names, synonyms, or IDs and suggest potential matches by auto-completion. These suggestions during typing the search string will be very helpful for users if the NCBI GeneID or the exact scientific name of the gene is not known.

With the Roche Cancer Genome Database we already cover a wide range of cancer related data and offer a convenient way to use this data in an integrated fashion. For the future, we will continue to integrate new external cancer related data into the warehouse system. The Roche Cancer Genome Database is publicly available at http://rcgdb.bioinf.uni-sb.de/MutomeWeb/.

## Competing interests

The authors declare that they have no competing interests.

## Authors' contributions

JK designed the database, implemented the importer and the user interface, and drafted the manuscript. DM, SK, and HB participated in the design of the user interface and tested biological use cases. HPL provides the hardware infrastructure and tested the complete system. All authors read and approved the final manuscript.

## Pre-publication history

The pre-publication history for this paper can be accessed here:

http://www.biomedcentral.com/1755-8794/4/43/prepub

## Supplementary Material

Additional file 1 **Supplementary Figure S1: Two examples for batch searches: On the top, the result page of a gene batch search for the members of the HER-family**. Here, the researcher used the synonym "her2" and the unknown gene "her" for the ERBB2 gene. At the bottom, the result page of the cell line batch search shows all mutations, mutated genes, as well as the "non-mutated" genes. Click here for file

Additional file 2 **Supplementary Figure S2: Two examples for smart searches: The "Mutation Status" smart search finds the mutations of the HER-family, KRAS, and BRAF in five different cell lines**. The resulting matrix is shown in the blue box. The "Pathway Enrichment Analysis" smart search determines for all KEGG pathways whether the number of mutated genes found in prostate carcinoma samples is statistically significant. Click here for file

Additional file 3 **Supplementary Figure S3: An advanced search for all mutations of EGFR or ERBB2 in breast samples, which are no cell lines**. The found mutations and simple statistics are shown at the bottom. Click here for file
