# Supplementary material for: The Roche Cancer Genome Database 2.0
Source: BMC Med Genomics. 2011 May 17;4:43. doi: 10.1186/1755-8794-4-43 (PMC3114700; doi:10.1186/1755-8794-4-43)
Supplement: Additional file 1 — Supplementary Figure S1: Two examples for batch searches: On the top, the result page of a gene batch search for the members of the HER-family. Here, the researcher used the synonym "her2" and the unknown gene "her" for the ERBB2 gene. At the bottom, the result page of the cell line batch search shows all mutations, mutated genes, as well as the "non-mutated" genes. [file 1755-8794-4-43-S1.PDF]

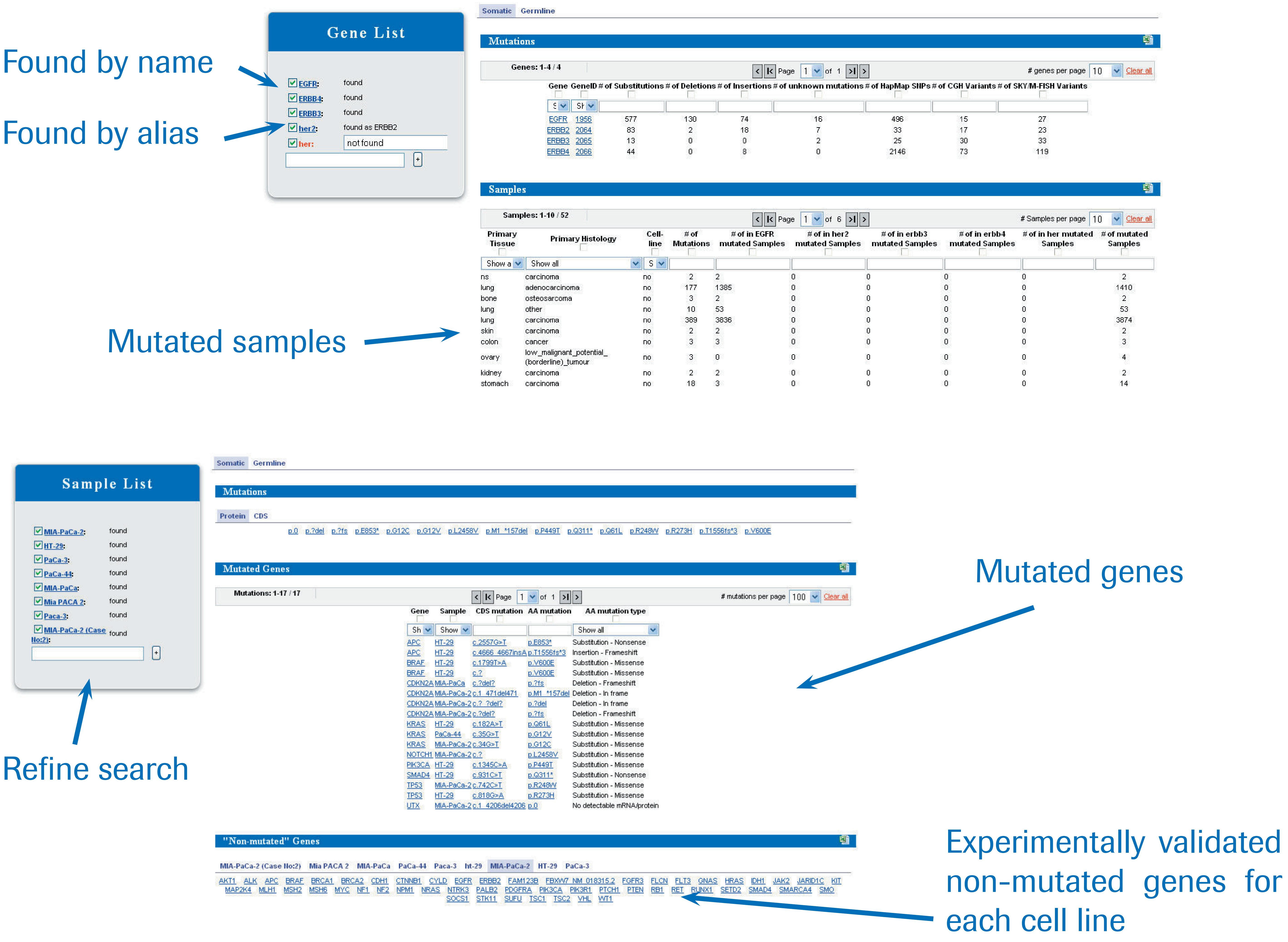
Found by name
Found by alias
Gene List
Mutated samples
Somatic
Germline
Mutations
Samples
Sample List
Refine search
Mutated Genes
Mutated genes
"Non-mutated" Genes
Experimentally validated non-mutated genes for each cell line
