# Supplementary material for: The Roche Cancer Genome Database 2.0
Source: BMC Med Genomics. 2011 May 17;4:43. doi: 10.1186/1755-8794-4-43 (PMC3114700; doi:10.1186/1755-8794-4-43)
Supplement: Additional file 2 — Supplementary Figure S2: Two examples for smart searches: The "Mutation Status" smart search finds the mutations of the HER-family, KRAS, and BRAF in five different cell lines. The resulting matrix is shown in the blue box. The "Pathway Enrichment Analysis" smart search determines for all KEGG pathways whether the number of mutated genes found in prostate carcinoma samples is statistically significant. [file 1755-8794-4-43-S2.PDF]

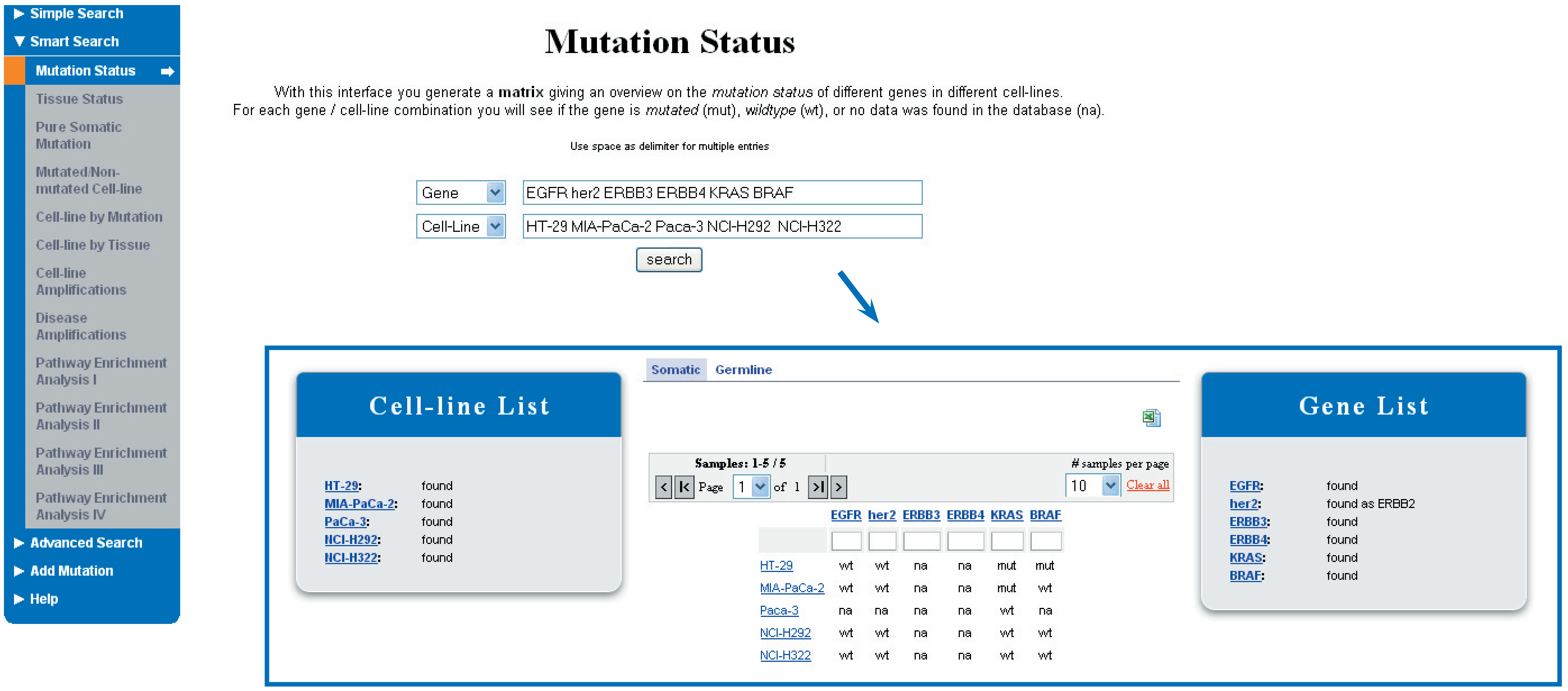
Simple Search
Smart Search
Mutation Status
Tissue Status
Pure Somatic Mutation
Mutated/Non-mutated Cell-line
Cell-line by Mutation
Cell-line by Tissue
Cell-line Amplifications
Disease Amplifications
Pathway Enrichment Analysis I
Pathway Enrichment Analysis II
Pathway Enrichment Analysis III
Pathway Enrichment Analysis IV
Advanced Search
Add Mutation
Help
Mutation Status
With this interface you generate a matrix giving an overview on the mutation status of different genes in different cell-lines.
For each gene / cell-line combination you will see if the gene is mutated (mut), wildtype (wt), or no data was found in the database (na).
Use space as delimiter for multiple entries
Gene
EGFR her2 ERBB3 ERBB4 KRAS BRAF
Cell-Line
HT-29 MIA-PaCa-2 Paca-3 NCI-H292 NCI-H322
search
Cell-line List
HT-29: found
MIA-PaCa-2: found
PaCa-3: found
NCI-H292: found
NCI-H322: found
Somatic
Germline
Samples: 1-5 / 5
samples per page
Page 1 of 1
10
Clear all
EGFR her2 ERBB3 ERBB4 KRAS BRAF
HT-29 wt wt na na mut mut
MIA-PaCa-2 wt wt na na mut wt
Paca-3 na na na na wt na
NCI-H292 wt wt na na wt wt
NCI-H322 wt wt na na wt wt
Gene List
EGFR: found
her2: found as ERBB2
ERBB3: found
ERBB4: found
KRAS: found
BRAF: found

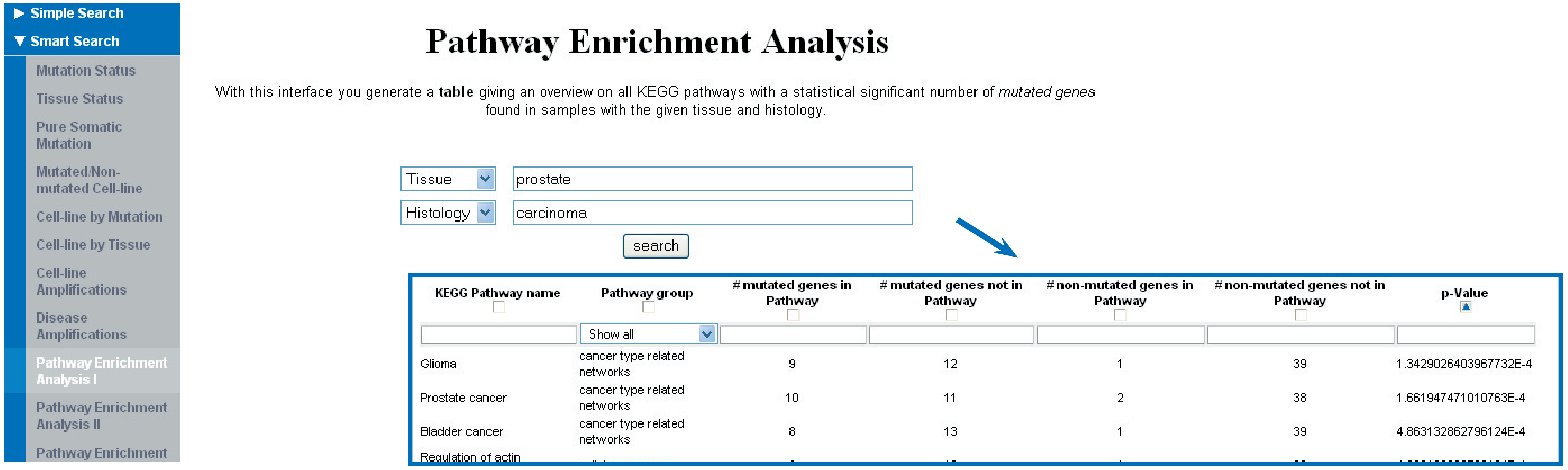
Simple Search
Smart Search
Mutation Status
Tissue Status
Pure Somatic Mutation
Mutated/Non-mutated Cell-line
Cell-line by Mutation
Cell-line by Tissue
Cell-line Amplifications
Disease Amplifications
Pathway Enrichment Analysis I
Pathway Enrichment Analysis II
Pathway Enrichment
Pathway Enrichment Analysis
With this interface you generate a table giving an overview on all KEGG pathways with a statistical significant number of mutated genes found in samples with the given tissue and histology.
Tissue
prostate
Histology
carcinoma
search
KEGG Pathway name
Pathway group
mutated genes in Pathway
mutated genes not in Pathway
non-mutated genes in Pathway
non-mutated genes not in Pathway
p-Value
Show all
Glioma | cancer type related networks | 9 | 12 | 1 | 39 | 1.3429026403967732E-4
Prostate cancer | cancer type related networks | 10 | 11 | 2 | 38 | 1.661947471010763E-4
Bladder cancer | cancer type related networks | 8 | 13 | 1 | 39 | 4.863132862796124E-4
Regulation of actin
