# Supplementary material for: The Roche Cancer Genome Database 2.0
Source: BMC Med Genomics. 2011 May 17;4:43. doi: 10.1186/1755-8794-4-43 (PMC3114700; doi:10.1186/1755-8794-4-43)
Supplement: Additional file 3 — Supplementary Figure S3: An advanced search for all mutations of EGFR or ERBB2 in breast samples, which are no cell lines. The found mutations and simple statistics are shown at the bottom. [file 1755-8794-4-43-S3.PDF]

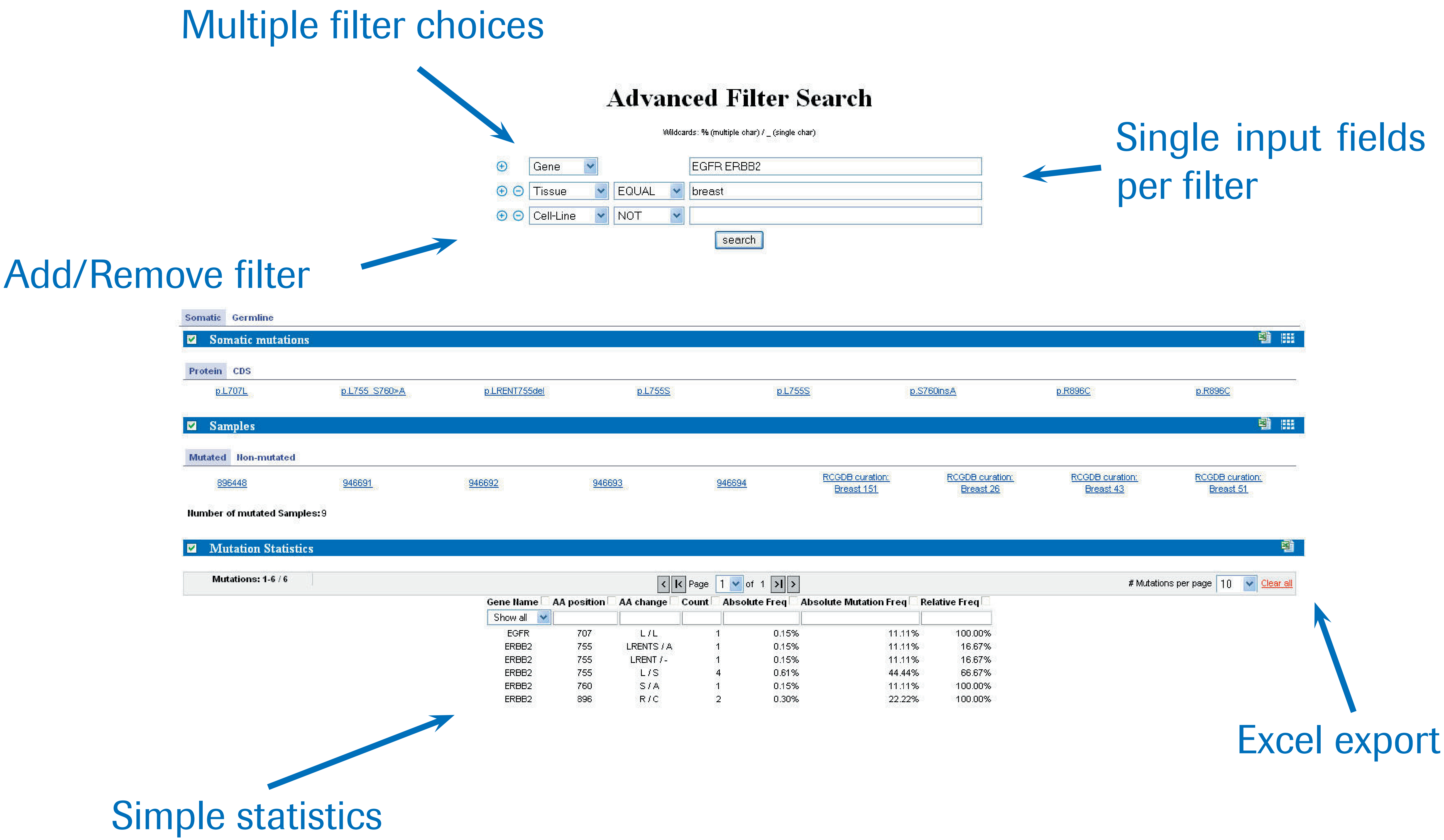

Gene Name	AA position	AA change	Count	Absolute Freq	Absolute Mutation Freq	Relative Freq
EGFR	707	L / L	1	0.15%	11.11%	100.00%
ERBB2	755	LRENTS / A	1	0.15%	11.11%	16.67%
ERBB2	755	LRENT / -	1	0.15%	11.11%	16.67%
ERBB2	755	L / S	4	0.61%	44.44%	66.67%
ERBB2	760	S / A	1	0.15%	11.11%	100.00%
ERBB2	896	R / C	2	0.30%	22.22%	100.00%
